# Short‐wave Infrared Photoluminescence Lifetime Mapping of Rare‐Earth Doped Nanoparticles Using All‐Optical Streak Imaging

**DOI:** 10.1002/advs.202305284

**Published:** 2024-01-06

**Authors:** Miao Liu, Yingming Lai, Miguel Marquez, Fiorenzo Vetrone, Jinyang Liang

**Affiliations:** ^1^ Centre Énergie Matériaux Télécommunications, Institut National de la Recherche Scientifique Université du Québec 1650 boulevard Lionel‐Boulet, Varennes Québec J3X1P7 Canada

**Keywords:** anti‐counterfeiting, lifetime‐based photoluminescence nanothermometry, rare‐earth doped nanoparticles, short‐wave infrared, ultrahigh‐speed imaging

## Abstract

The short‐wave infrared (SWIR) photoluminescence lifetimes of rare‐earth doped nanoparticles (RENPs) have found diverse applications in fundamental and applied research. Despite dazzling progress in the novel design and synthesis of RENPs with attractive optical properties, existing optical systems for SWIR photoluminescence lifetime imaging are still considerably restricted by inefficient photon detection, limited imaging speed, and low sensitivity. To overcome these challenges, SWIR photoluminescence lifetime imaging microscopy using an all‐optical streak camera (PLIMASC) is developed. Synergizing scanning optics and a high‐sensitivity InGaAs CMOS camera, SWIR‐PLIMASC has a 1D imaging speed of up to 138.9 kHz in the spectral range of 900–1700 nm, which quantifies the photoluminescence lifetime of RENPs in a single shot. A 2D photoluminescence lifetime map can be acquired by 1D scanning of the sample. To showcase the power of SWIR‐PLIMASC, a series of core‐shell RENPs with distinct SWIR photoluminescence lifetimes is synthesized. In particular, using Er^3+^‐doped RENPs, SWIR‐PLIMASC enables multiplexed anti‐counterfeiting. Leveraging Ho^3+^‐doped RENPs as temperature indicators, this system is applied to SWIR photoluminescence lifetime‐based thermometry. Opening up a new avenue for efficient SWIR photoluminescence lifetime mapping, this work is envisaged to contribute to advanced materials characterization, information science, and biomedicine.

## Graphical abstract

Short‐wave infrared (SWIR) photoluminescence lifetime imaging microscopy using an all‐optical streak camera (PLIMASC) reaches a 1D imaging speed of up to 138.9 kHz in the 900–1700 nm spectral range. SWIR‐PLIMASC is implemented to map lifetimes of downshifting emission by rare‐earth doped nanoparticles with applications in anti‐counterfeiting and thermometry.

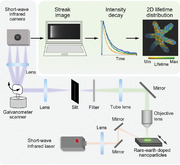

## 1 Introduction

Rare‐earth doped nanoparticles (RENPs) have provoked widespread curiosity for their applicability in a plethora of fields, including nanomedicine, bioimaging, security authentication, and sensing.^[^ ^1^ ^]^ Their popularity is mostly attributed to their diverse photoluminescence properties that span multiple excitation wavelengths and emission possibilities.^[^ ^2^ ^]^ Of significant interest, many RENPs can be excited by short‐wave infrared (SWIR) light (also referred to as near‐infrared light). In biomedicine, SWIR excitation light (in the biological windows^[^ ^3^ ^]^) mitigates the drawbacks associated with UV and/or visible light excitation, such as strong scattering by tissue and large absorption of biomolecules, which enables deep tissue imaging.^[^ ^4^ ^]^ Invisible to the naked eye, SWIR light is also well‐suited for defense and surveillance.^[^ ^5^ ^]^ The emission of RENPs presents remarkable properties as well. After multiphoton excitation, RENPs can convert the SWIR light to the UV and visible ranges, a process known as upconversion.^[^ ^6^ ^]^ Meanwhile, via single‐photon excitation, RENPs can also emit in the SWIR range through downshifting emission,^[^ ^7^ ^]^ which has better penetration capability compared to the upconverting counterpart and avoids interference of background light. Thus, RENPs, which are excited in the SWIR region and can simultaneously emit in the UV, visible, and SWIR ranges, offer an attractive nanoplatform for both fundamental and applied research.

Among the SWIR optical properties of RENPs, the photoluminescence lifetime has recently ignited a surge of interest. The lifetime is an intrinsic characteristic of rare‐earth photoluminescence that provides valuable information regarding the photophysical properties of the RENPs, e.g., energy transfer processes (Note S1, Supporting Information) and the photoluminescence efficiency.^[^ ^8^ ^]^ In practice, photoluminescence lifetime does not vary with the concentration of RENPs and the penetration depth. It is minimally affected by many circumstances even the exterior conditions.^[^ ^9^ ^]^ Circumventing many problems in intensity‐based measurements, photoluminescence lifetime imaging and sensing enables a reliable detection method,^[^ ^9^, ^10^ ^]^ which is particularly beneficial for accuracy‐demanding applications, such as secured information storage and temperature monitoring.^[^ ^11^ ^]^ Meanwhile, the study of photoluminescence lifetime can, in turn, aid in the understanding of the luminescence process and, thus, in the design of new RENPs with improved optical properties, including a high quantum yield and a tunable photoluminescence lifetime.^[^ ^12^ ^]^

Existing photoluminescence lifetime imaging techniques still encounter various limitations in the SWIR range. The most common technique is time‐correlated single‐photon counting (TCSPC) using an InGaAs single‐photon avalanche diode (SPAD).^[^ ^13^ ^]^ Despite having a high signal‐to‐noise ratio (SNR), this method requires a large number of repeated excitations to the same location because the detector can only process a limited number of photons for each excitation. The long SWIR photoluminescence lifetimes of RENPs (i.e., from hundreds of microseconds to several milliseconds) restrict the excitation's repetition rate, which results in much extended pixel dwelling time to build the photoluminescence intensity decay curve. To accelerate data acquisition, many technical innovations have explored spatial parallelism and real‐time (i.e., the time during which an event occurs) detection.^[^ ^14^ ^]^ The former strategy is represented by the invention of SPAD arrays^[^ ^15^ ^]^ and the performance enhancement of SWIR CCD/CMOS cameras.^[^ ^16^ ^]^ Nonetheless, SPAD arrays confront the same limitations as their point‐detection counterpart.^[^ ^17^ ^]^ SWIR CCD/CMOS cameras enjoy a high detection sensitivity. However, their pixel structures and readout mechanisms inevitably limit their frame rates to ≈100 frames per second (fps), falling short of directly recording the dynamics of SWIR emission on the microsecond time scale. The required speed can be provided by image‐converter streak cameras, which convert time to space by deflecting photoelectrons to different spatial locations.^[^ ^18^ ^]^ Although capable of recording photoluminescence intensity decay in real time using a single excitation pulse, these instruments have exceptionally low sensitivity because the low energy of SWIR photons diminishes the generation of photoelectrons in the photocathode.^[^ ^19^ ^]^ Thus far, imaging techniques have not kept up to provide technical specifications tailored for highly efficient SWIR photoluminescence lifetime acquisition.

To surmount this challenge, we develop SWIR photoluminescence lifetime imaging microscopy using an all‐optical streak camera (PLIMASC), which combines scanning optics with a high‐sensitivity InGaAs CCD camera for 1D ultrahigh‐speed imaging at a speed of up to 138.9 kHz. By 1D scanning of the sample, SWIR‐PLIMASC maps the 2D SWIR photoluminescence lifetime distribution. To evaluate the system and assess its potential in two important applications, a series of Er^3+^ (or Ho^3+^)‐doped RENPs with distinct SWIR photoluminescence decay lifetimes are prepared. The SWIR‐PLIMASC system is applied to multiplexed SWIR anti‐counterfeiting using the Er^3+^‐doped RENPs while it is implemented in SWIR photoluminescence lifetime‐based thermometry using the Ho^3+^‐RENPs as nanothermometers.

## 2 Results

### 2.1 Preparation of RENPs

**Figure 1:**
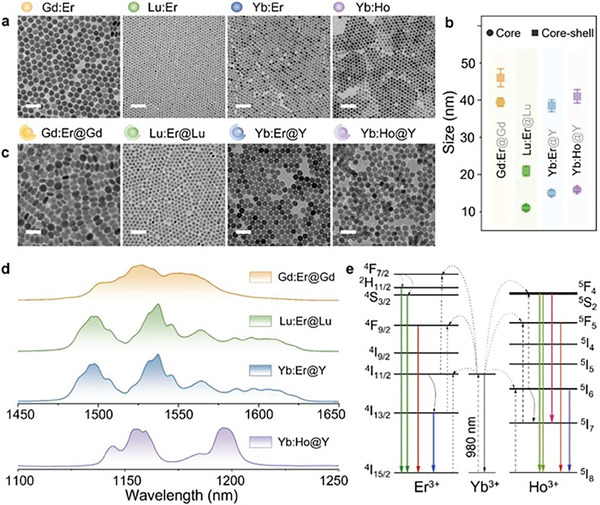
Morphology and downshifting photoluminescence of the RENPs under investigation. a) Transmission electron microscopy images of four types of core RENPs. b) Size distributions of all the core and core‐shell RENPs. c) Same as (a), but showing core‐shell RENPs. d) SWIR photoluminescence spectra of the four types of core‐shell RENPs under 980 nm excitation. e) Energy level diagrams of Er^3+^‐doped RENPs and Ho^3+^‐doped RENPs under 980 nm excitation. Scale bar in (a,c): 100 nm.

To tune the photoluminescence lifetimes of RENPs, we varied the host material, particle size, shell thickness, and dopant (activator) ions. All RENPs (i.e., Er^3+^ and Ho^3+^‐doped RENPs) were synthesized using the thermal decomposition method previously described.^[^ ^20^ ^]^ First, a series of Er^3+^ (2 mol%)‐doped RENPs were prepared and their photoluminescence lifetimes were tuned by changing the host matrix from NaGdF_4_ to LiLuF_4_ and to LiYbF_4_. The Yb^3+^ ion was selected as the sensitizer (to increase 980 nm photon absorption),^[^ ^21^ ^]^ and the concentration was kept at 18 mol% (in the case of LiLuF_4_ and NaGdF_4_ where it is added as a co‐dopant) while for LiYbF_4_, the Yb^3+^ sensitizer was part of the host matrix. The first three panels in **Figure** **1**a show the transmission electron microscopy (TEM) images of the core NaGdF_4_:Yb^3+^, Er^3+^, LiLuF_4_:Yb^3+^, Er^3+^, and LiYbF_4_:Er^3+^ RENPs, respectively. All the core RENPs are uniform and monodispersed with estimated diameters to be ≈40, 11, and 15 nm, respectively (Figure 1b). Subsequently, the luminescent core RENPs were coated with an inert shell to minimize the surface defects as well as external quenching and ultimately increase the photoluminescence intensity. The final core‐shell structures shown in the first three panels in Figure 1c are NaGdF_4_:Yb^3+^, Er^3+^@NaGdF_4_ (Gd:Er@Gd), LiLuF_4_:Yb^3+^, Er^3+^@LiLuF_4_ (Lu:Er@Lu), and LiYbF_4_:Er^3+^@LiYF_4_ (Yb:Er@Y), respectively. The shell thickness varies from 3, 5, and 11 nm, respectively (see Figure S1, Supporting Information).^[^ ^20^, ^22^ ^]^ For further analysis of these core‐shell RENPs, their crystal phases were examined using X‐ray diffraction (XRD) measurements (Figure S2, Supporting Information). All the diffraction peaks matched well with the standard diffraction patterns.

Under 980 nm laser excitation, the three Er^3+^‐doped core‐shell RENPs showed SWIR emission centered at ≈1535 nm (Figure 1d), corresponding to the ^4^I_13/2_ → ^4^I_15/2_ transition. The energy level diagram (showing the excited states of interest) is shown in Figure 1e. Amongst all the various processes to populate the Er^3+^ excited states, the most dominant one likely involves Yb^3+^ to Er^3+^ energy transfer. In particular, excited Yb^3+^ ions transfer their absorbed energy to the adjacent Er^3+^ ions that are subsequently excited to the ^4^I_11/2_ state via ground state absorption. The emitting ^4^I_13/2_ state is populated after a multiphonon relaxation process from the ^4^I_11/2_ state, leading to the following SWIR emission. The SWIR spectra of Lu:Er@Lu and Yb:Er@Y materials are similar—both spectra present multiple fine emission peaks. In contrast, the Gd:Er@Gd RENPs show broadband emission, which is consistent with previous studies.^[^ ^23^ ^]^ The multiple emission peaks in the LiLuF_4_ or LiYbF_4_ samples are attributed to the pronounced Stark splitting of Er^3+^ in these matrices, which exert high crystal field strengths on the dopants.^[^ ^23^, ^24^ ^]^ Alongside the downshifting emission, upconversion emissions at ≈520/540 nm and 660 nm were also observed (Figure S3, Supporting Information), which are assigned to the ^2^H_11/2_ / ^4^S_3/2_ → ^4^I_15/2_ and ^4^F_9/2_ → ^4^I_15/2_ transitions, respectively.

Another activator ion, Ho^3+^, was also investigated as a dopant for the RENPs. While its photoluminescence properties in the visible region (upconversion) are similar to Er^3+^, its emission in the SWIR is different, which can be used to showcase the system's viability. Specifically, we synthesized LiYbF_4_:Ho^3+^ core and LiYbF_4_:Ho^3+^@LiYF_4_ (Yb:Ho@Y) core‐shell RENPs. The TEM images (the last panels in Figure 1a,c) show uniform nanoparticles with distributions of ≈16 and 41 nm in diameter (Figure 1b) for the core and core‐shell structures, respectively. The crystal structure was confirmed by the XRD measurement (Figure S2, Supporting Information). The downshifting photoluminescence spectrum of Yb:Ho@Y was collected, as shown in Figure 1d. The SWIR emission at ≈1154 nm is attributed to the Ho^3+ 5^I_6_ → ^5^I_8_ transition. Moreover, the upconversion emissions at ≈540, 650, and 750 nm were also observed (Figure S3, Supporting Information), assigned to the radiative transitions of ^5^S_2_/^5^F_4_ → ^5^I_8_, ^5^F_5_ → ^5^I_8_, and ^5^S_2_/^5^F_4_ → ^5^I_7_, respectively.

### 2.2 Operating Principle of SWIR‐PLIMASC

**Figure 2:**
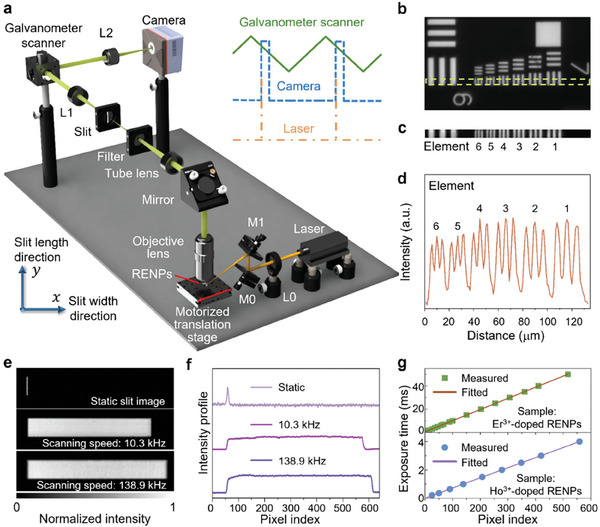
Short‐wave infrared photoluminescence lifetime imaging microscopy using an all‐optical streak camera (SWIR‐PLIMASC). a) Schematic. M0–M1, Mirror; L0–L2, Lens. Inset: Diagram of synchronization of the galvanometer scanner's triangular control signal (green solid line), the camera's exposure (blue dashed line), and the laser pulses (orange dash‐dotted line). b) Reference image of a USAF resolution target. c) Local line‐scanning image of Group 7 elements on the USAF resolution target. d) Averaged intensity profile of Element 1–6 of Group 7 in (c). e) Static image and streak images of the slit under two different imaging configurations. f) Averaged intensity profiles of the slit images in (e). g) Linearity test at two imaging speeds. RENPs: Rare‐earth doped nanoparticles.

The schematic of the SWIR‐PLIMASC is shown in **Figure** **2**a. A 980 nm laser, controlled by an external trigger, generates microsecond‐level pulses for illumination. A 200‐mm‐focal‐length plano‐convex lens (Thorlabs, LA1708‐B‐ML, marked as L0) focuses the pulse onto the sample plane. The visible and SWIR emissions of the RENPs are collected by a 20× objective lens (Mitutoyo, 378‐824‐16, 0.4 numerical aperture). The light passes through a mirror, a tube lens, and a band‐pass filter (Semrock BLP01‐1064R‐25). In this way, both the upconversion emission and the back‐scattered excitation light are filtered out, and an image of the object with the targeted SWIR emission band is formed at the intermediate image plane. There, a slit (100 µm × 2 mm, Fineline Imaging) is placed to limit the field of view to one dimension. Then, the slit image is relayed to a SWIR CMOS camera (Xenics, Cheetah‐640CL TE3) by a 4*f* imaging system composed of two 100‐mm‐focal‐length lenses (Thorlabs, AC254‐100‐C, marked as L1 and L2 in Figure 2a). A galvanometer scanner (GS), placed at the Fourier plane and driven by a triangular wave, temporally shears the dynamic slit images along its width (i.e., *x*) direction. Thus, each image captured by the camera records the *y*‐*t* information within the exposure time. The 1D imaging speed, denoted by γ_1_, is determined by

(1) $$\gamma_{1}=\frac{\gamma V_{g}f_{2}}{t_{\mathrm{tri}}d}$$

Here, γ is a constant that links the deflection angle and the voltage of the input waveform on GS (i.e., *V*_g_).^[^ ^25^ ^]^ *f*_2_ = 100 mm is the focal length of lens L2. *t*_tri_ is the period of the triangular waveform. *d*  = 20 µm is the pixel size of the camera. In addition, the exposure time, denoted by *t*_e_, determines the number of time bins in each *y*‐*t* plot by *N_t_* = *r*_l_ *t*_e_. Finally, the sample is scanned in the *x* direction by a motorized translation stage (Physik Instrumente, L‐509.10SD00) synchronized with the laser, the GS, and the camera (see the inset of Figure 2a). It is worth noting that the frequency of the trigger on the laser is the same as on the camera to ensure there is only one pulse excitation during the exposure time. In this way, SWIR‐PLIMASC efficiently records the full photoluminescence intensity decay in a single shot.

The system performance of SWIR‐PLIMASC was characterized by the following three aspects. First, to assess the system's spatial resolution, we covered the Gd:Er@Gd core‐shell RENPs with a negative USAF resolution target (Edmund Optics, 55–622). Figure 2b,c shows a reference image (i.e., a wide‐field snapshot with no slit) and the local line‐scanning result of Group 7 with the slit. The averaged intensity profile of Element 1–6 of the line‐scanning result is plotted in Figure 2d, showing good contrast for all elements. The spatial resolution, quantified by analyzing an edge spread function (Figure S4, Supporting Information), was determined to be 2.27 µm, which closely matched the theoretical value. Second, to accommodate the varied photoluminescence lifetimes of the RENPs, the imaging speed and time bins of SWIR‐PLIMASC were tuned by changing the period and the voltage of the triangular waveform applied to the GS as well as the exposure time of the camera. In particular, for Er^3+^‐doped RENPs, *t*_tri_ =  200 ms, *V*_g_ =  3 V, and *t*_e_ =  50 ms. For Ho^3+^‐doped RENPs, *t*_tri_ =  50 ms, *V*_g_ =  10 V, and *t*_e_ =  4 ms. Figure 2e shows the static image of the slit and the streak images of the slit under these two configurations from steady emission under continuous‐wave 980 nm excitation. The intensity profiles of the slit in Figure 2e, averaged in the *y* direction, are plotted in Figure 2f, which yielded *r*_l_ = 10.3 kHz (corresponding to 97.6 µs pixel^−1^) for Er^3+^‐doped RENPs and *r*_l_ = 138.9 kHz (corresponding to 7.2 µs pixel^−1^) for Ho^3+^‐doped RENPs. Finally, to examine the linearity in the temporal shearing operation, we varied the exposure time from 0.2 to 50 ms and recorded the width of the streak images. The results were fitted with a linear function (Figure 2g). The slopes of the fitted curves were quantified to be 10.4 and 139.8 kHz. The deviation was attributed to pixel counting error and different shutter responses for various exposure times, which exerted a larger impact for higher imaging speed. For the ensuing experiment, the exposure times were set to 50 ms for the Er^3+^‐doped RENPs and 4 ms for the Ho^3+^‐doped RENPs.

SWIR‐PLIMASC presents several attractive advantages in characterizing the photoluminescence lifetime of the RENPs. First, the system is responsive to a wide spectral range from 900 to 1700 nm and thus photoluminescence at different wavelengths and/or spectral bands can be detected by changing the band‐pass filter. To the best of our knowledge, SWIR‐PLIMASC marks the first of its kind in high‐sensitivity, ultrahigh‐speed SWIR imaging. Second, the system offers high tunability in 1D imaging speeds from 10.3 to 138.9 kHz. Benefiting from this ability, SWIR photoluminescence lifetimes from microseconds (µs) to milliseconds (ms) can be directly captured using the same system. Moreover, the shearing operation allocates temporal information in photoluminescence to different spatial positions, so that the entire process of the 1D photoluminescence intensity decay can be recorded in a snapshot. Circumventing the dead time in photon processing, SWIR‐PLIMASC has a higher light throughput compared with the TCSPC and SPAD techniques.

### 2.3 Anti‐Counterfeiting Using SWIR‐PLIMASC

**Figure 3:**
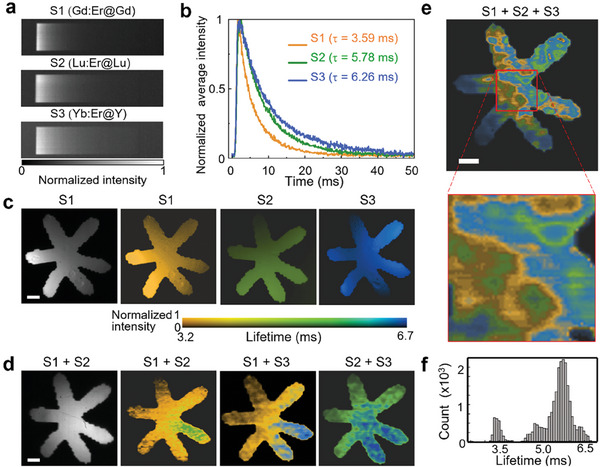
Photoluminescence lifetime‐based anti‐counterfeiting using SWIR‐PLIMASC with Er^3+^‐doped core‐shell RENPs. a) Streak images of three types of Er^3+^‐doped core‐shell RENPs, denoted by S1–S3. b) Normalized average photoluminescence intensity decay curves of S1–S3 plotted from the corresponding images in (a). c) Intensity image and photoluminescence lifetime images of the three types of Er^3+^‐doped core‐shell RENPs covered by the transparency of a flower. d) Photoluminescence lifetime images of the mixture of two types of RENPs. e) Photoluminescence lifetime image of mixed three types of RENPs with a zoom‐in view of a local area. f) Histogram of photoluminescence lifetimes in the flower pattern shown in (e). Scale bar in (c,d,e): 50 µm.

Anti‐counterfeiting has become an essential practice to protect valuable commodities.^[^ ^26^ ^]^ Among existing techniques, luminescent RENPs are becoming widely studied as advanced anti‐counterfeiting materials due to their attractive properties, such as excellent photostability and high compatibility with printing techniques.^[^ ^27^ ^]^ While photoluminescence spectrum‐based anti‐counterfeiting coding has a lower security level due to the well‐defined and known positions of RENPs’ spectral bands, photoluminescence lifetime‐based anti‐counterfeiting patterns offer better protection owing to its transience in emission, fine tunability, and demand of specialized examination tools. Storing encrypted information in the time domain, photoluminescence lifetime‐based anti‐counterfeiting can avoid photoluminescence overlap in the spectrum and background interference.^[^ ^28^ ^]^ Thus, using SWIR‐PLIMASC, we explored anti‐counterfeiting by SWIR photoluminescence lifetime mapping of RENPs. Under a 1D imaging speed of *r*_l_ =  10.3 kHz, the streak images of the three types of Er^3+^‐doped core‐shell RENPs are shown in **Figure** **3**a. By averaging the intensity in the *y* direction, the normalized photoluminescence intensity decay curves are plotted in Figure 3b. The photoluminescence lifetime was extracted by synthetically considering the finite width of the slit and the finite duration of the excitation pulse (Note S2 and Figure S5, Supporting Information). Using this method, the photoluminescence lifetimes (denoted by τ) at ≈1535 nm in Gd:Er@Gd, Lu:Er@Lu, and Yb:Er@Y RENPs were calculated to be 3.59, 5.78, and 6.26 ms, respectively. Then, we covered these RENP samples with a transparency of a flower. Their photoluminescence lifetime maps are shown in Figure 3c, and the histograms of photoluminescence lifetimes in each case are shown in Figure S6 (Supporting Information).

These Er^3+^‐doped core‐shell RENPs, which had similar photoluminescence spectra but different photoluminescence lifetimes, provided temporal codes for SWIR anti‐counterfeiting. First, we mixed two of the three different types of RENPs. A different type of RENP was used in one or two petals of the flower pattern to encrypt the spatially heterogeneous photoluminescence lifetimes (Figure 3d). While the intensity image, as a camouflage, shows similar brightness of these petals with the surrounding ones (see the first panel of Figure 3d as well as Figure S7, Supporting Information), SWIR‐PLIMASC reveals the encrypted content—the different photoluminescence lifetimes in the designated locations. To further test SWIR‐PLIMASC's ability to decrypt complex photoluminescence lifetime information, we mixed all three types of RENPs dispersed in hexane. A drop containing S1 was deposited and dried on the glass slide. Using this protocol, the second and third drops containing S2 and S3 were applied to the same location. The 2D photoluminescence lifetime distribution of this sample is shown in Figure 3e. Interestingly, the complex photoluminescence lifetime distribution resembles water stains, reflecting the flow and drying of the hexane solvent during sample preparation. Finally, by analyzing these 2D photoluminescence lifetime images (e.g., Figure 3d,e), the photoluminescence lifetime histogram in each sample was extracted (see Figure 3f; Figure S8, Supporting Information). Compared with the histograms in Figure S6 (Supporting Information), the broadened ranges and larger overlaps of photoluminescence lifetimes in the histogram in Figure 3f were likely attributed to the mixture of the RENPs. Considering the difficult‐to‐reproduce nature of the all‐mixed sample, SWIR‐PLIMASC is poised to accurately decrypt complex photoluminescence lifetime‐coded information. Benefiting from this delicate topology composed of only three types of RENPs, this anti‐counterfeiting design shows potential for a SWIR photoluminescence lifetime‐based taggant with a high‐security level.

### 2.4 Photoluminescence Thermometry Using SWIR‐PLIMASC

The Boltzmann‐coupled emission bands in RENPs are highly sensitive to temperature changes.^[^ ^29^ ^]^ Leveraging this photoluminescence property, RENPs, as temperature indicators, were teamed up with advanced imaging modalities for remote‐detection, minimally invasive, and high‐resolution photoluminescence thermometry.^[^ ^30^ ^]^ These advances inspired us to apply SWIR‐PLIMASC to photoluminescence lifetime‐based thermometry. In particular, the large energy mismatch between Yb^3+^ (^2^F_5/2_) and Ho^3+^ (^5^I_6_) provides a strong temperature‐induced variation of the phonon‐assisted energy transfer from Yb^3+^ to Ho^3+^,^[^ ^31^ ^]^ making Yb:Ho@Y RENPs ideal sensitive temperature indicators for SWIR photoluminescence lifetime‐based thermometry.

**Figure 4:**
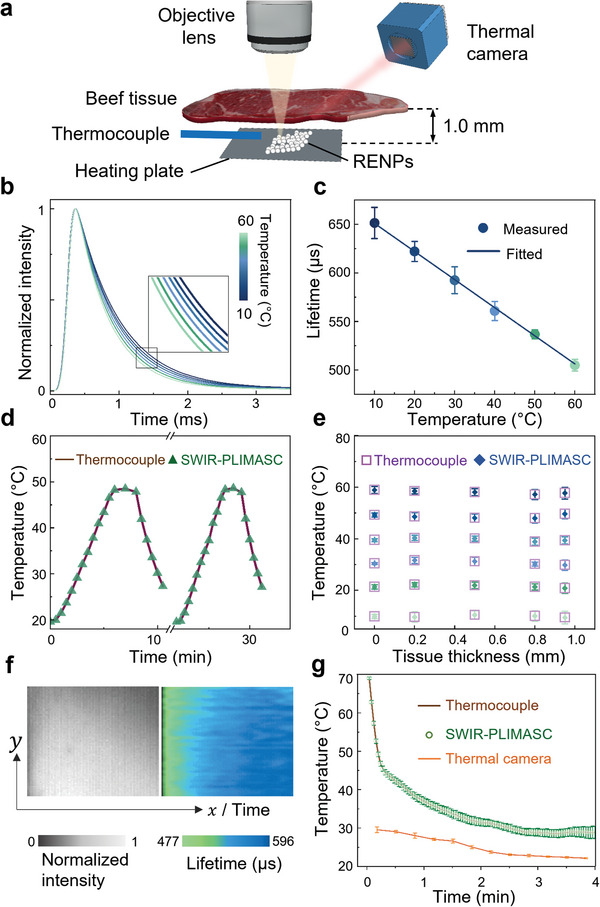
Photoluminescence thermometry using SWIR‐PLIMASC with LiYbF_4_:Ho^3+^@LiYF_4_ RENPs. a) Schematic representation of the experimental setup. b) Normalized photoluminescence intensity decays of RENPs from 10 to 60 °C. c) Temperature dependence of the average photoluminescence lifetime of RENPs with linear fitting. d) Longitudinal temperature monitoring. e) Temperature sensing from 10 to 60 °C underneath beef tissue with different thicknesses. f) Dynamic temperature mapping through 0.5 mm‐thick beef tissue during the cooling of the heating plate. g) Comparison of temperature sensing ability of SWIR‐PLIMASC with the thermal camera. Error bars in (c,e,g): standard deviation.

The experimental setup is shown in **Figure** **4**a. The temperature of the sample was controlled by a heating plate and double‐checked by a Type K thermocouple (Omega, HH306A). SWIR‐PLIMASC was applied with a 1D imaging speed of *r*_l_ =  138.9 kHz. In the proof‐of‐concept experiment, SWIR‐PLIMASC captured the intensity decay of the Ho^3+^‐doped core‐shell RENPs without scattering medium (Figure S9, Supporting Information). The same analysis applied to the experiments of Er^3+^‐doped RENPs was used here to extract the photoluminescence lifetime of Yb:Ho@Y RENPs. As shown in Figure 4b, the photoluminescence lifetime monotonically decreases with temperature from 651.4 µs at 10 °C to 505.0 µs at 60 °C, which is fitted with a linear curve with a slope of *S*_a_ = −2.9 µs°C^−1^ and an intercept of 680.0 µs, where *S*_a_ denotes the absolute temperature sensitivity (Figure 4c). Moreover, we calculated the relative sensitivity *S*_r_ (Figure S10, Supporting Information) and quantified the measured lifetime uncertainty of the optical system (0.32–1.29% at room temperature). The details are provided in Note S3 (Supporting Information). Finally, to validate the reliability of this technique, we conducted a longitudinal monitoring of temperature cycled between 20 and 50 °C for over 30 min. As shown in Figure 4d, the measured temperatures are in good agreement with the temperature change measured by the thermocouple as the gold standard.

To test SWIR‐PLIMASC in a biological environment, a piece of beef tissue with a varied thickness up to 0.94 mm as the absorbing and scattering medium was placed at 1.0 mm above the sample. As shown in Figure 4e, SWIR‐PLIMASC can accurately image temperature distribution beneath biological tissue. Moreover, we recorded the cooling process of the sample covered by 0.5 mm‐thick beef tissue. The temperature of the sample was first increased to ≈70 °C. Then, the heating plate was turned off. The SWIR‐PLIMASC system continuously captured 80 images to record the photoluminescence lifetime/temperature change. Besides SWIR‐PLIMASC, a thermal camera was also used to monitor the temperature evolution. The lifetime/temperature map captured by SWIR‐PLIMASC is shown in Figure 4f. The averaged curve is plotted in Figure 4g, which shows a drastic temperature decrease from 70 to 37 °C in the first minute, followed by a much slower decrease to 29 °C as the sample approaches room temperature in 4 min. The result also shows excellent agreement with the temperature measured by the thermocouple. In contrast, the measurement from the thermal camera shows considerable errors of up to 40 °C. Detached from the sample, the covered beef tissue was not heated up but was almost kept at room temperature. Only capable of surface temperature sensing, the thermal camera failed to report the temperature of RENPs located beneath the beef tissue. The biological phantom in this experiment served as an analogy to the temperature difference between the skin and the core body. The accurate temperature sensing showcases SWIR‐PLIMASC's advantage over thermal camera‐based temperature measurement, indicating the prospect of deep tissue temperature monitoring for potential applications in early cancer theranostics.

## 3 Discussion

In summary, we develop SWIR‐PLIMASC—an efficient photoluminescence lifetime mapping platform specialized for RENPs in the SWIR spectral range. The implementation of scanning optics endows the InGaAs CMOS camera with a 1D ultrahigh imaging speed of up to 138.9 kHz while allowing it to retain its high sensitivity in the SWIR spectral range, which enables efficient quantification of RENPs’ SWIR photoluminescence lifetimes. Combined with the Er^3+^‐doped RENPs, this technique reveals complex topology in photoluminescence lifetime‐based taggants, which shows great potential in high‐security anti‐counterfeiting. Using Ho^3+^‐doped RENPs that present strong temperature dependence, we implement SWIR‐PLIMASC in accurate temperature sensing in a biological phantom, demonstrating its superior detection ability compared to conventional thermal imaging.

In the experiments, we have taken the following precautions to minimize the impacts of various photonic effects on the measurement accuracy in SWIR‐PLIMASC. First, the results could be affected by noises (e.g., shot noise and readout noise) and background.^[^ ^32^ ^]^ To reduce the impact of the shot noise, we optimized the excitation laser power for high SNR acquisition. To mitigate the readout noise, we used 14‐bit quantization, a moderate gain in data acquisition, and a relatively low readout bandwidth. We also leveraged three‐stage thermoelectric cooling implemented on the camera. To avoid the background, we performed the experiments in a dark room and covered the beam path to eliminate the stray light. Second, the results could be influenced by the local density of optical states (LDOS).^[^ ^33^ ^]^ Nonetheless, we used thick RENP layers as the sample, which should largely average any photonic distortions induced by the varying LDOS. Moreover, neither a reflector nor inhomogeneity was present in the vicinity of RENPs to confound the results. Finally, temperature was exacted from the intensity decay of emission, not by the intensity ratio of two emission bands. Thus, considering the photonic artifacts induced by SWIR‐emitting RENPs in ratiometric thermometry, SWIR‐PLIMASC presents a more suitable modality to accommodate these RENPs for accurate measurement, which can thus fully leverage their deeper penetration advantages in biomedical applications.^[^ ^32^, ^33^, ^34^ ^]^

The consideration of these photonic effects points to attractive future research directions. First, they could guide the system design and equipment selection to minimize the photonic distortions. Meanwhile, they would be further leveraged in in situ calibration for the temperature readout in an inhomogeneous environment.^[^ ^34^, ^35^ ^]^ Finally, they could propel the design and fabrication of innovative RENPs with tunable SWIR photoluminescence lifetimes. All these efforts will aid SWIR‐PLIMASC, as an advanced characterization technique, for broad applications in information science and biomedicine.

## 4 Experimental Section

### Preparation of RENPs Precursors

Stoichiometric amounts of RE_2_O_3_ (RE = Lu, Gd, Yb, Er, Ho) were selectively mixed with 5 mL trifluoroacetic acid and 5 mL distilled water in a 50 mL three‐neck round bottom flask. The mixture was kept at 80 °C under vigorous stirring until the solution became clear. The temperature was then reduced to 60 °C to evaporate the residual trifluoroacetic acid and water.

### Synthesis of NaGdF_4_:18% Yb^3+^, 2% Er^3+^@NaGdF_4_

NaGdF_4_:18% Yb^3+^, 2% Er^3+^ core RENPs were synthesized via the hot injection thermolysis approach. Specifically, the precursor (CF_3_COO)_3_RE (RE = Gd/Yb/Er) and CF_3_COONa were dispersed in Solution A0 [containing 7.5 mL of 1‐octadecene (ODE) and 7.5 mL of oleic acid (OA)] and degassed at 125 °C under vigorous stirring. Then, Solution A0 was injected into the preheated mixture of 12.5 mL of OA and 12.5 mL of ODE (Solution B0) at a rate of 1.5 mL min^−1^ (Harvard Apparatus Pump 11 Elite). The final solution was kept at 315 °C under an argon atmosphere for 1 h to obtain the core RENPs. The core‐shell structure was prepared by epitaxial growth of the shell on the preformed cores. 0.5 mmol of core RENPs were mixed with 10 mL of OA and 10 mL of ODE (Solution A1). Shell (2 mmol) precursors (CF_3_COO)_3_Gd together with CF_3_COONa were mixed with 10 mL of OA and 10 mL of ODE (Solution B1). Solution A1 and B1 were both degassed at 110 °C for 30 min. After degassing, Solution A1 was heated to 290 °C. Solution B1 was injected into Solution A1 at a 0.75 mL min^−1^ rate when the temperature of Solution A1 was stable. After cooling down to room temperature, the final product was washed with hexane/ethanol (1:3) three times and re‐dispersed in hexane.

### Synthesis of LiYbF_4_:18% Yb^3+^, 2% Er^3+^/Ho^3+^@LiYF_4_ and LiLuF_4_:18% Yb^3+^, 2% Er^3+^@LiLuF_4_

These RENPs were prepared via a two‐step thermal decomposition method from the first nuclei to the core RENPs. A mixture of 7 mL of OA, 7 mL of oleylamine (OM), and 14 mL ODE (Solution A2) was degassed at 110 °C for 15 min and then heated to 330 °C under an argon atmosphere. Meanwhile, 2.5 mmol CF_3_COOLi and (CF_3_COO)_3_RE (RE = Lu/Yb, Er/Ho) were mixed with 3 mL OA and 6 mL ODE (Solution B2) and then degassed at 125 °C for 30 min. OM (3 mL) was added in Solution B2 and left to degas for 5 min. Once the temperature of Solution A2 was stable, Solution B2 was injected into Solution A2 with a rate of 1.5 mL min^−1^. After a 1 h reaction at 330 °C, the preparation of the first nuclei was finished. Core RENPs were formed by stabilizing first nuclei with an excess of OA. 1.25 mmol first nuclei were mixed with 16 mL of OA and 16 mL of ODE in a 100 mL three‐neck round bottom flask. The solution was degassed at 110 °C for 30 min and backfilled with argon gas. The temperature was raised to 315 °C, after which the reaction was continued for 1 h.

Core‐shell RENPs were prepared by using the same method mentioned above except for the rare‐earth source. The only difference was the reaction time. After each ≈7 mL injection of solution B1 to solution A1, the mixture was allowed to react for 40 min.

## Conflict of Interest

The authors declare no conflict of interest.

## Supporting information

Supporting Information

## Data Availability

The data that support the findings of this study are available from the corresponding author upon reasonable request.
