# Supplementary material for: Short‐wave Infrared Photoluminescence Lifetime Mapping of Rare‐Earth Doped Nanoparticles Using All‐Optical Streak Imaging
Source: Adv Sci (Weinh). 2024 Jan 6;11(11):2305284. doi: 10.1002/advs.202305284 (PMC10953585; doi:10.1002/advs.202305284)
Supplement: Supplementary file 1 — Supporting Information [file ADVS-11-2305284-s001.pdf]

ADVANCED SCIENCE
Open Access

Supporting Information

for *Adv. Sci.*, DOI 10.1002/advs.202305284

Short-wave Infrared Photoluminescence Lifetime Mapping of Rare-Earth Doped Nanoparticles Using All-Optical Streak Imaging

Miao Liu, Yingming Lai, Miguel Marquez, Fiorenzo Vetrone and Jinyang Liang**

Supporting Information

Short-wave infrared photoluminescence lifetime mapping of rare-earth doped nanoparticles using all-optical streak imaging

Miao Liu, Yingming Lai, Miguel Marquez, Fiorenzo Vetrone, Jinyang Liang**

Centre Énergie Matériaux Télécommunications, Institut National de la Recherche Scientifique, Université du Québec, 1650 boulevard Lionel-Boulet, Varennes, Québec J3X 1P7, CANADA

Corresponding authors: Fiorenzo.Vetrone@inrs.ca (F.V.), Jinyang.Liang@inrs.ca (J.L.)

Materials

Gd_2O_3 (REacton 99.999%), Lu_2O_3 (REacton, 99.999%), Yb_2O_3 (REacton, 99.998%), Er_2O_3 (REacton, 99.99%), Ho_2O_3 (REacton, 99.997%), 1-octadecene (ODE, 90%), and oleic acid (OA, 90%) were purchased from Alfa Aesar (USA). Lithium trifluoroacetate (98%), sodium trifluoroacetate (98%), oleylamine (OM, 70%), and trifluoroacetic acid (TFA, 99%) were purchased from Sigma-Aldrich. All chemicals were used as received.

Characterization

Structural Characterization. The crystallinity and phases of the core and core-shell rare-earth doped nanoparticles (RENPs) were determined via X-ray powder diffraction analysis on a Bruker D8 Advance Diffractometer (USA) using Cu Kα radiation (λ=1.5418 Å). The morphology and size distribution of the core and core-shell structures were further investigated by transmission electron microscopy (TEM, Philips Tecnai 12, USA). The particle size was determined from TEM images using ImageJ software with a minimal set size of 100 particles.

Optical Characterization. Upconversion and downshifting spectra of oleate-capped RENPs in hexane were obtained at room temperature under the excitation of a 980-nm laser (MDL-SN-980-10W, CNI, China). Laser power and power density were about 490 mW and 8.0 W/cm^2, respectively. All spectra were measured in a quartz cuvette of 1 cm optical path, filled with 2 mL of RENPs at 10 mg/mL concentration. The upconversion emission was recorded with an Avaspec-ULS2048L spectrometer (Avantes, The Netherlands). Stray light from the excitation source was

removed with an 830-nm short-pass filter. The downshifting emission was collected with a Shamrock 500i monochromator (Andor, Ireland) equipped with an iDus InGaAs 1.7 NIR detector (Andor, Ireland). A 980-nm long-pass filter was used to remove any stray light from the excitation source.

Supplementary Note 1: Background of the photoluminescence lifetime of rare-earth doped nanoparticles

The photoluminescence lifetime of RENPs, denoted by τ, is determined by

$$\tau = \frac{1}{W_{\mathrm{R}} + W_{\mathrm{NR}}}. \quad (S1)$$

Here, W_{R} is the radiative transition rate, and W_{NR} refers to the non-radiative transition rate.

According to the Judd-Ofelt theory, the rate of relaxation from an initial state J to the final state J' through the radiative transition is determined by [1]

$$W_{\mathrm{R}}(J, J') = \frac{64\pi^2\Delta^3}{3h(2J+1)}(\chi\bar{F}^2 + n^3\bar{M}^2). \quad (S2)$$

Here, Δ is the energy gap between J and J'. h is the Planck constant. $\chi = n(n^2+2)^2/9$ is the Lorentz correction for the local field with n being the refractive index of the host material. $\bar{F}^2$ and $\bar{M}^2$ are the matrix elements of the electric dipole and magnetic dipole moments, respectively [2].

The non-radiative transition rate W_{NR} is related to temperature T via the Arrhenius equation [3]

$$W_{\mathrm{NR}} \propto \exp(-\Delta E/kT). \quad (S3)$$

Here, ΔE is the energy gap between the lowest level of the excited states and a possible non-radiative decay state. k is the Boltzmann constant.

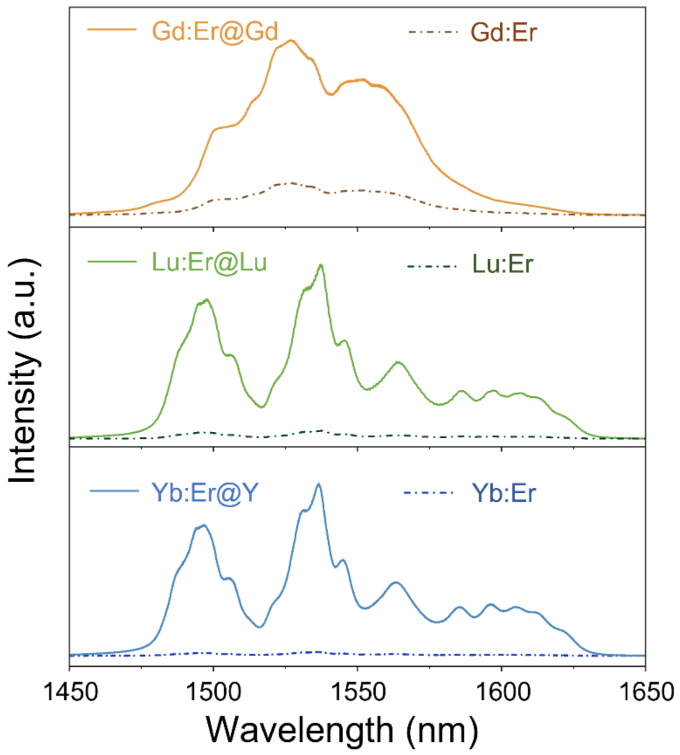

Figure S1. Short-wavelength infrared (SWIR) spectra of the core and the corresponding core-shell RENPs under 980-nm excitation. $NaGdF_4$:Yb^{3+}, Er^{3+} (Gd:Er); $NaGdF_4$:Yb^{3+}, Er^{3+}@$NaGdF_4$ (Gd:Er@Gd); $LiLuF_4$:Yb^{3+}, Er^{3+} (Lu:Er); $LiLuF_4$:Yb^{3+}, Er^{3+}@$LiLuF_4$ (Lu:Er@Lu); $LiYbF_4$:Er^{3+} (Yb:Er); $LiYbF_4$:Er^{3+}@$LiYF_4$ (Yb:Er@Y).

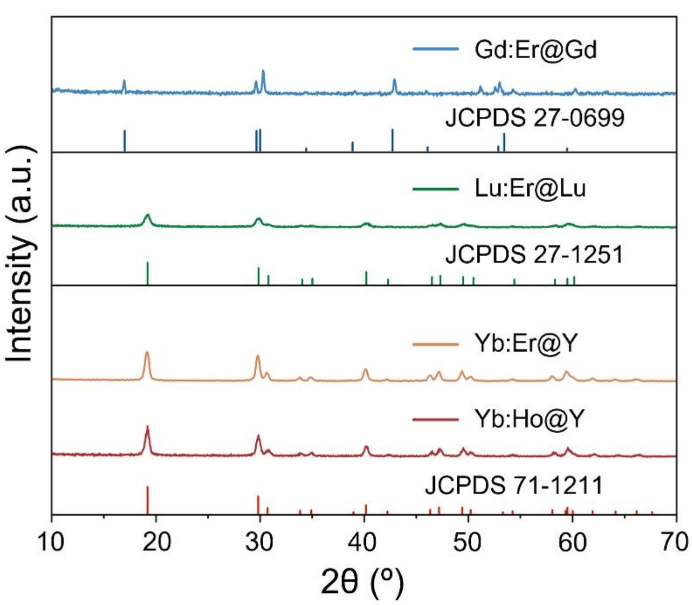

Figure S2. X-ray diffraction patterns of the core-shell RENPs. $NaGdF_4$:Yb^{3+}, Er^{3+}@$NaGdF_4$ (Gd:Er@Gd); $LiLuF_4$:Yb^{3+}, Er^{3+}@$LiLuF_4$ (Lu:Er@Lu); $LiYbF_4$:Er^{3+}@$LiYF_4$ (Yb:Er@Y); $LiYbF_4$:Ho^{3+}@$LiYF_4$ (Yb:Ho@Y).

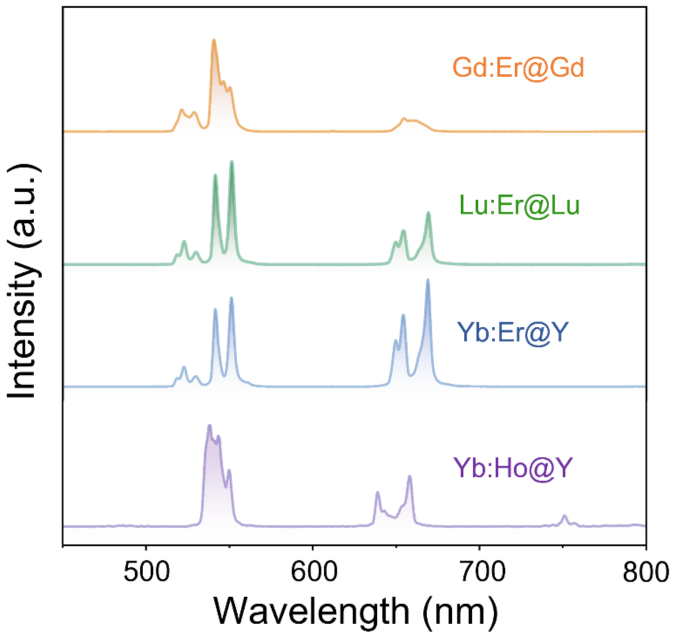

Figure S3. Upconversion photoluminescence spectra of core-shell RENPs under 980-nm excitation. $NaGdF_4$:Yb^{3+}, Er^{3+}@$NaGdF_4$ (Gd:Er@Gd); $LiLuF_4$:Yb^{3+}, Er^{3+}@$LiLuF_4$ (Lu:Er@Lu); $LiYbF_4$:Er^{3+}@$LiYF_4$ (Yb:Er@Y); $LiYbF_4$:Ho^{3+}@$LiYF_4$ (Yb:Ho@Y).

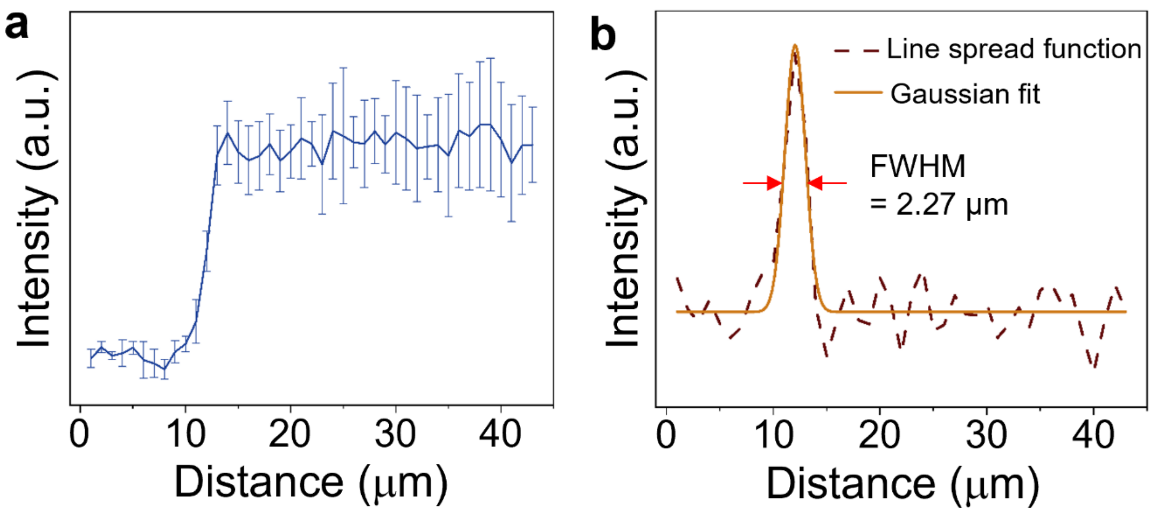

Figure S4. Quantification of spatial resolution of SWIR photoluminescence lifetime imaging microscopy using an all-optical streak camera (PLIMASC). (a) Edge spread function extracted from an edge on a USAF resolution target. Error bar: standard deviation. **(b)** Line spread function produced from (a) with a Gaussian fit. FWHM: Full width at half maximum.

Supplementary Note 2: Determination of the photoluminescence lifetime of the RENP sample

The determination of photoluminescence lifetimes synthetically considers the excitation pulse profile [denoted by $I_{\mathrm{pulse}}(t)$] and the finite width of the slit [denoted by $I_{\mathrm{slit}}(t)$]. In particular, the measured intensity decay can be deduced from the convolution

$$I(t) = I_{\mathrm{pulse}}(t) * I_{\mathrm{slit}}(t) * O(t), \tag{S4}$$

where $O(t) = \exp(-t/\tau)$ represents the mono-exponential function with a lifetime of τ. In this work, $I_{\mathrm{pulse}}(t)$ is measured by a photodiode, and $I_{\mathrm{slit}}(t)$ is extracted from the static slit image. Then, $I(t)$ is computed and used to fit the experimental result.

Figure S5 shows the photoluminescence lifetime extraction. The excitation pulse width was measured to be 200 μs. $I_{\mathrm{pulse}}(t)$ was produced after filtering the noise and cleaning the background (Figure S5a). In addition, the static slit image was averaged in the y direction (see an example in Figure 2e in Main Text) to produce a line profile (Figure S5b). Using the shearing relation [i.e., 97.6 μs/pixel (see Section 2.2 in Main Text)], the spatial pixels were converted to time bins, which produced $I_{\mathrm{slit}}(t)$. As an example, the normalized average photoluminescence intensity decay curve of Gd:Er@Gd (see Figure 3b in Main Text) and the fit are plotted in Figure

S5c. Its corresponding semi-log plot is shown in Figure S5d. The lifetime of Gd:Er@Gd was determined by the fitted $I(t)$ that gave the highest R-squared value. For this sample, its photoluminescence lifetime was determined to be 3.59 ms (with $R^2 = 0.9821$). Finally, the semi-log plots of the normalized average photoluminescence intensity decay of Lu:Er@Lu, and Yb:Er@Y RENPs (see Figure 3b in Main Text) are shown in Figures S5e–f.

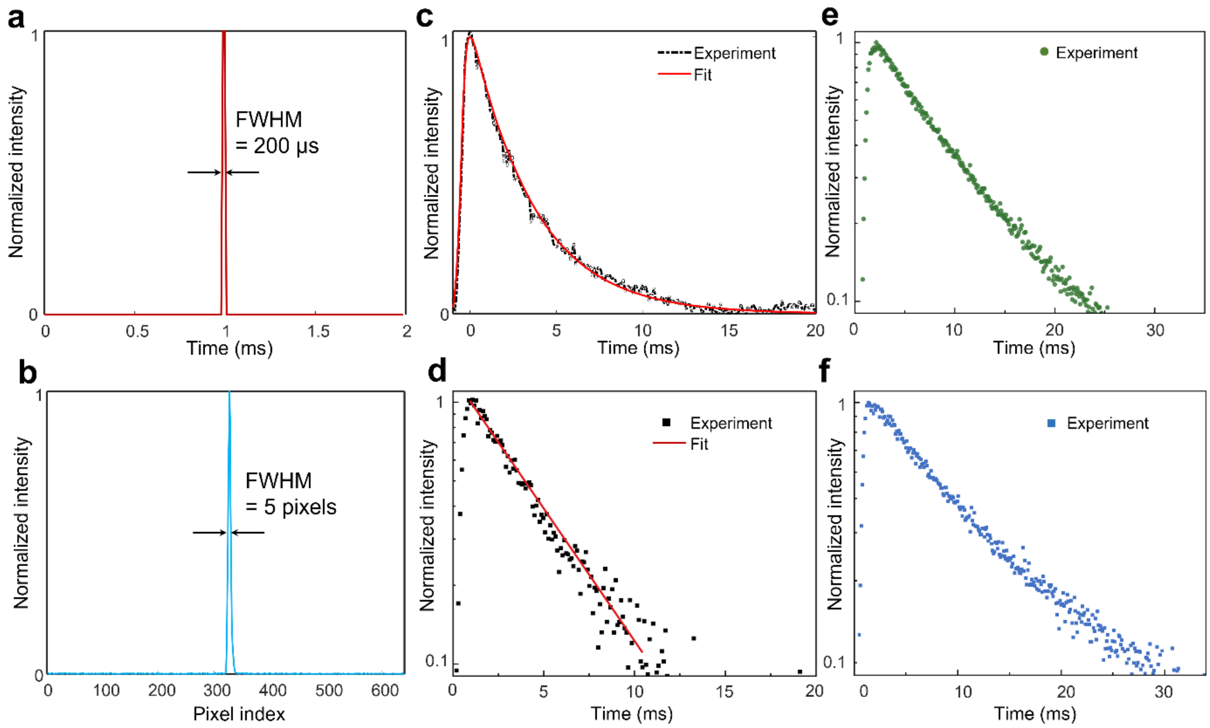

Figure S5. Determination of photoluminescence lifetime for Gd:Er@Gd RENP samples. (a) Normalized excitation pulse profile. **(b)** Averaged spatial profile of the slit. **(c)** Measured photoluminescence intensity decay of Gd:Er@Gd with the fit. **(d)** As (c), but plotted in the logarithmic scale. **(e)–(f)** As (d), but showing the results of Lu:Er@Lu (e) and Yb:Er@Y (f).

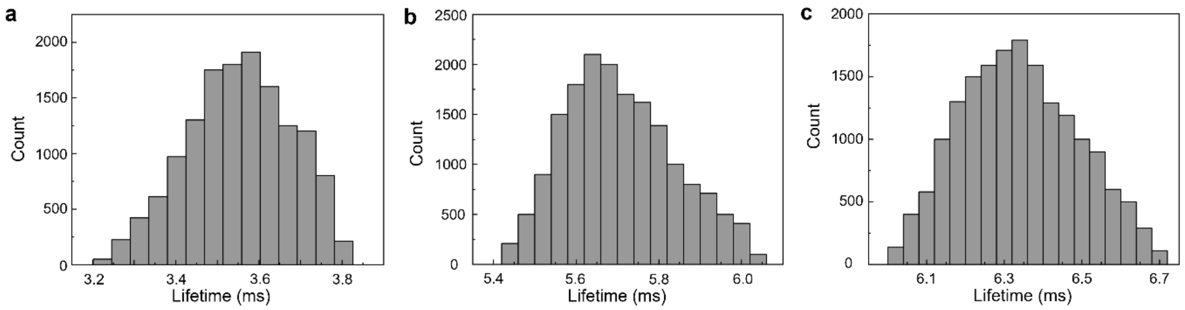

Figure S6. Photoluminescence lifetime histograms of RENPs corresponding to Figure 3c. (a) Gd:Er@Gd (i.e., S1). **(b)** Lu:Er@Lu (i.e., S2). **(c)** Yb:Er@Y (i.e., S3).

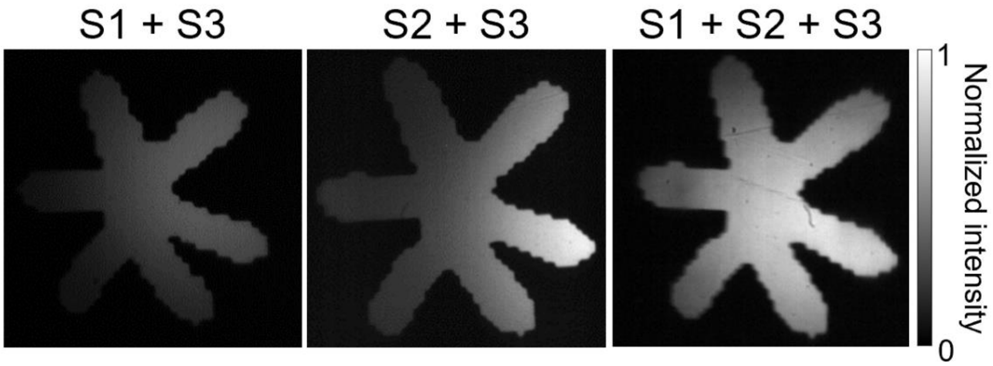

Figure S7. SWIR intensity images of the samples composed of the mixture of Gd:Er@Gd (i.e., S1), Lu:Er@Lu (i.e., S2), and Yb:Er@Y (i.e., S3) RENPs.

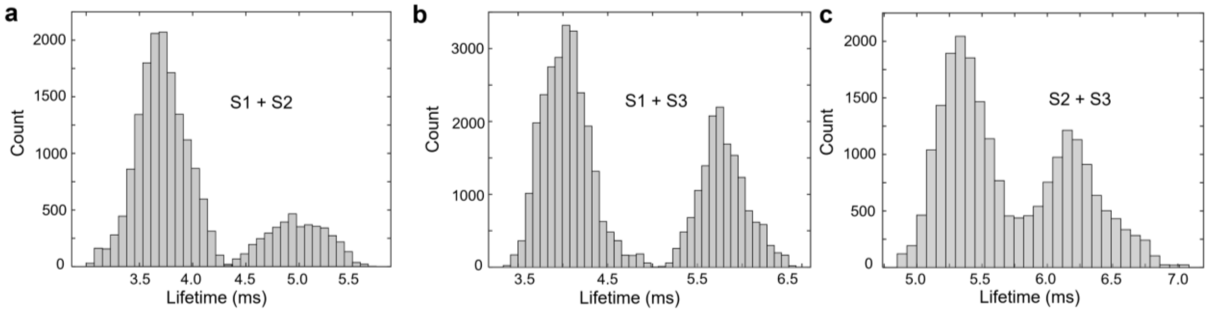

Figure S8. Photoluminescence lifetime histograms of RENPs corresponding to Figure 3d. (a) S1 + S2. **(b)** S1 + S3. **(c)** S2 + S3.

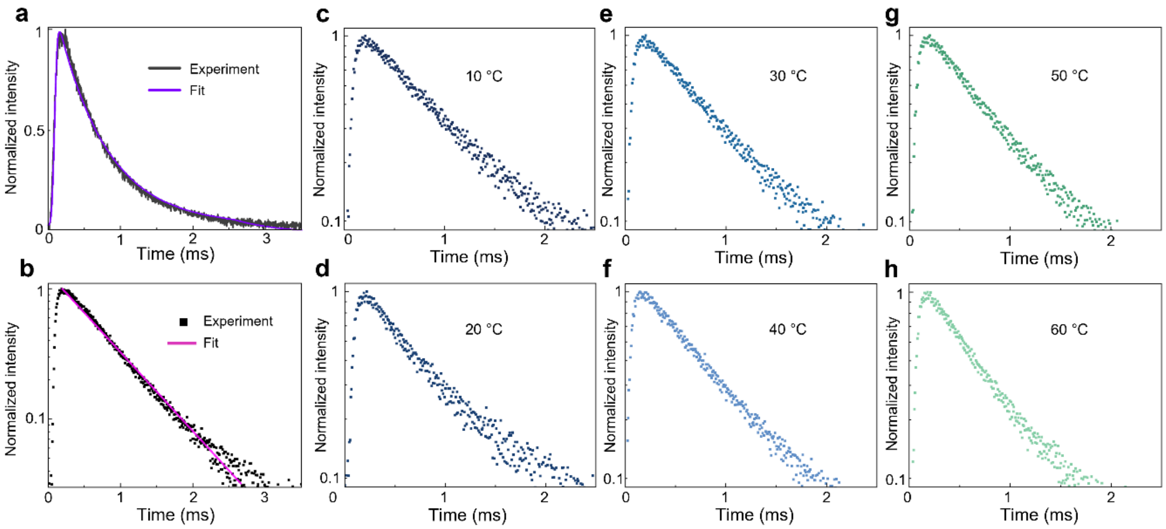

Figure S9. Photoluminescence intensity decay of $LiYbF_4$:Ho^{3+}@$LiYF_4$ RENPs. (a) Normalized average intensity decay curve at room temperature with the fit. **(b)** As (a), but plotted in the logarithmic scale. **(c-h)** As (b), but showing the photoluminescence intensity decays at six different temperatures.

Supplementary Note 3: Performance of photoluminescence lifetime-based luminescence thermometry

Relative sensitivity. The photoluminescence lifetime-based relative sensitivity S_r is calculated as follows:

$$S_\mathrm{r} = \left|\frac{1}{\tau}\frac{\partial\tau}{\partial T}\right|. \quad \text{(S5)}$$

Here, S_r demonstrates the percentage change of the photoluminescence lifetime per unit temperature.

Thermal uncertainty. Thermal uncertainty δT is calculated by

$$\delta T = \frac{1}{S_\mathrm{r}}\frac{\delta\tau}{\tau}. \quad \text{(S6)}$$

Equation (S6) shows that the temperature uncertainty of the RENPs is influenced by the performance of the nanoparticles themselves and the photoluminescence lifetime fluctuation measured by the optical system. In this work, the standard deviation of the lifetimes was quantified to be 3.86 μs in 40 repeated measurements.

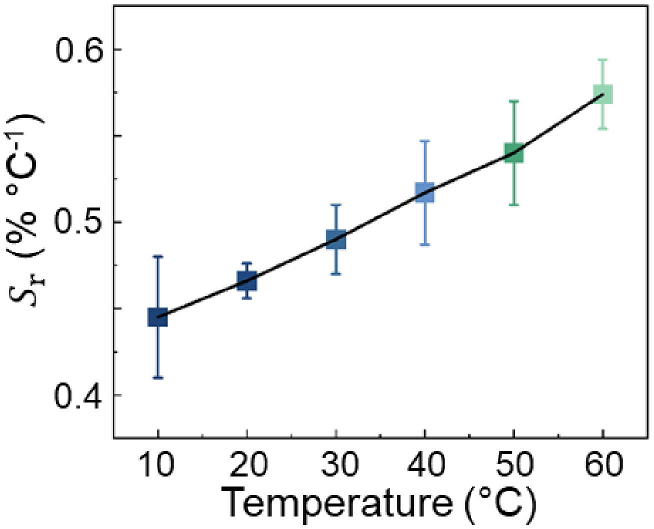

Figure S10. Relative sensitivity of $LiYbF_4$:Ho^{3+}@$LiYF_4$ RENPs.

Supplementary References

[1] G. Liu, *Chem. Soc. Rev.* **2015**, 44, 1635.

[2] a) B. R. Judd, *Phys. Rev.* **1962**, 127, 750; b) G. S. Ofelt, *J. Chem. Phys.* **2004**, 37, 511; c) M. P. Hehlen, M. G. Brik, K. W. Krämer, *J. Lumin.* **2013**, 136, 221.

[3] a) L. A. Riseberg, H. W. Moos, *Phys. Rev.* **1968**, 174, 429; b) X.-d. Wang, O. S. Wolfbeis, R. J. Meier, *Chem. Soc. Rev.* **2013**, 42, 7834.
